# *In vivo* selection of novel biotherapeutics

**DOI:** 10.18632/oncotarget.5247

**Published:** 2015-08-22

**Authors:** Giulia Ruozi, Mauro Giacca

**Affiliations:** Molecular Medicine Laboratory, International Centre for Genetic Engineering and Biotechnology (ICGEB), Trieste, Italy

**Keywords:** Chromosome Section, AAV vectors, biological therapeutics, secretome, regeneration

There is an impelling demand to develop novel therapeutics against degenerative disorders. Approximately 30% of people over 80 years of age develop a dementia and 1-3% of those over 65 years have Parkinson’s disease. Over 17 million patients suffer from heart failure, mostly as a consequence of lack of cardiac regeneration after ischemic cardiomyocyte death. After 75 years of age, 30% of individuals undergo age-related macular degeneration, a major cause of irreversible blindness, and 50% develop presbycusis.

All these degenerative conditions have at least three features in common: first, the function and structure of the affected tissues deteriorate over time; second, the functional cells in these organs (cardiomyocytes, neurons, retinal cells, neuroepithelial cells of the inner ear) are incapable of significant regeneration in adult life; third, there are no effective therapies slowing the progressive loss of these cells and, even less, promoting their regeneration.

Despite the lack of curative therapies, notable progress has been achieved in understanding the cellular and molecular mechanisms leading to tissue degeneration. Thus, there is hope that novel biological therapeutics, able to specifically interfere with the different molecular mechanisms of disease onset and progression, might offer therapeutic opportunities. How to identify these therapeutics, however, remains a formidable challenge.

The screening of genetic libraries for a given function directly *in vivo* (thus moving from biochemical or phenotypic selection *in vitro* towards functional selection *in vivo*) would represent an exceptional asset for the identification of novel therapeutic factors, in the absence of any a priori, possibly biased, information on their molecular function. In contrast to phenotypic selection, however, functional screening of genetic libraries for degenerative conditions is still in its infancy, especially due to the intrinsic difficulty of delivering genes to post-mitotic tissues.

Indeed, functional selection *in vivo* demands a gene delivery vector that i) can be produced at very high titers; ii) infects resting or post-mitotic cells very efficiently, and iii) persists in these cells for prolonged periods of time in the absence of significant transgene silencing. Vectors based on the Adeno-Associated Virus (AAV) appear to suit all these requirements. These are relatively small vectors (viral capsid ∼20 nm, genome size < 5 kb) that transduce post-mitotic cells *in vivo* at high efficiency, such as heart, brain and retina, and drive persistent transgene expression in these organs [1].

Our recent work [2] reports on the development of an innovative procedure for *in vivo* functional selection, which we named FunSel, in which arrayed AAV vector gene collections are used to iteratively transduce a given tissue to find potential therapeutic leads. A pool of AAV vectors, each one coding for a specific factor, is injected *in vivo* to transduce permissive cells (e.g. cardiomyocytes, skeletal muscle fibers, retinal cells, pancreas β-cells); each vector enters a different cell. After a few weeks, a selective stimulus is applied (e.g. coronary or femoral artery ligation, or chemical induction of cell death). Then, vector cDNA inserts are recovered and used to generate a new AAV preparation, which is submitted to additional cycles of selection. This iterative procedure leads to the selection of viral transgenes exerting a protective or regenerative effect on the expressing tissues (Figure 1).

**Figure 1:**
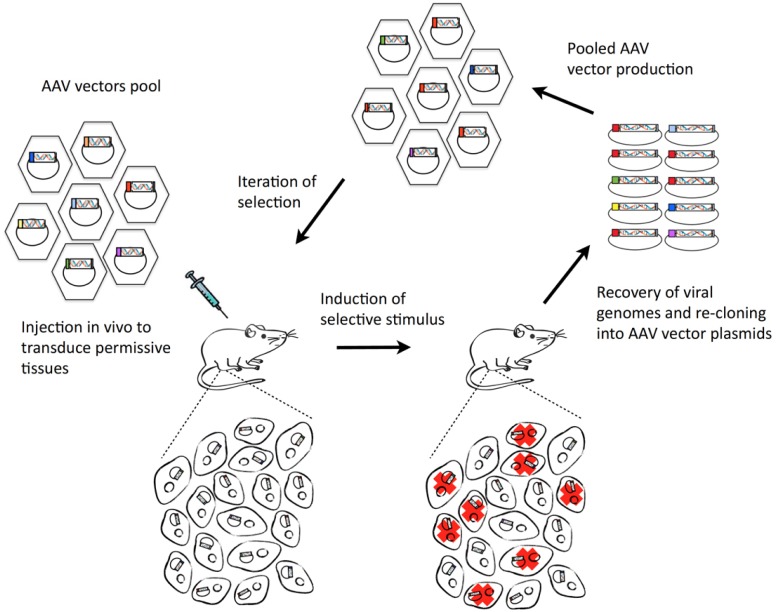
Outline of the AAV-based *in vivo* functional selection procedure (FunSel) for the identification of novel biological therapeutics for degenerative disorders Cf. text for description.

To provide proof-of-principle evidence of FunSel efficacy, we screened 100 factors from the murine secretome (including various cytokines, growth factors, hormones and extracellular matrix proteins) for their capacity to protect skeletal muscle and heart against acute ischemia. Using the analysed set of genes, FunSel led to the identification of ghrelin as a powerful myoprotective factor. Ghrelin is a 28-amino acid circulating hormone, physiologically produced by the stomach in conditions of starvation, and exerting a powerful secretagogue function eventually leading to the release of growth hormone from the pituitary [3]. Indeed we found that both the major circulating form of the peptide, which is octanoylated on serine 3 [4], and the unmodified peptide expressed from our AAV vectors, protect both skeletal muscle fibers and cardiomyocytes from ischemic cell death and preserve cardiac function over time.

Why was ghrelin selected by our functional screening? In principle, the tissue protective functions selected by FunSel could ensue from different molecular mechanisms, including inhibition of apoptosis, induction of neovascularization, protection from inflammation or immune response, among others. We found that a major function of ghrelin was to inhibit apoptosis through the induction of autophagy, a process exerting a fundamental role in both normal homeostasis and after tissue insult. In particular, autophagy specifically protects from ischemic damage by removing dysfunctional organelles through the lysosomal degradative pathway [5]. Under stressful conditions, particularly in the heart [6], autophagy is induced as an adaptive response to avoid cell death.

In principle, FunSel can be applied using arrayed genetic collections encoding for any kind of proteins, ranging from transcription factors to membrane receptors. The use of genetic libraries encoding for genes from the secretome (approximately 2,000 genes [7]) offers the important advantage of permitting the identification of factors that could eventually exert their therapeutic function once administered as recombinant proteins. This appears to be an important step towards the generation of new injectable, biological drugs targeting degenerative conditions.

## References

[1] Zacchigna S (2014). Circ Res.

[2] Ruozi G (2015). Nat Commun.

[3] Kojima M (1999). Nature.

[4] Yang J (2008). Cell.

[5] Mizushima N (2011). Cell.

[6] Kanamori H (2011). Cardiovasc Res.

[7] Grimmond SM (2003). Genome Res.

